# Selection Bias Risk in Randomized Controlled Trials Rated as Low Bias Using Risk of Bias, Version 2 (RoB2) Tool

**DOI:** 10.7759/cureus.63581

**Published:** 2024-07-01

**Authors:** Steffen Mickenautsch, Veerasamy Yengopal

**Affiliations:** 1 Faculty of Dentistry, University of the Western Cape, Cape Town, ZAF; 2 Community Dentistry, University of the Witwatersrand, Johannesburg, Johannesburg, ZAF

**Keywords:** risk of bias 2 tool, selection bias, i2, clinical trial, bias testing, systematic review

## Abstract

Our study aimed to establish the risk of selection bias in randomized controlled trials (RCT) that were overall rated as having “low bias” risk according to Cochrane’s Risk of Bias, version 2 (RoB 2) tool. A systematic literature search of current systematic reviews of RCTs was conducted. From the identified reviews, RCTs with overall “high bias” and “low bias” RoB 2 risk ratings were extracted. All RCTs were statistically tested for selection bias risk. From the test results, true positive, true negative, false positive, or false negative ratings were established, and the false omission rate (FOR) with a 95% confidence interval (CI) was computed. Subgroup analysis was conducted by computing the negative likelihood ratio (-LR) concerning RoB 2 domain 1 ratings: bias arising from the randomization process. A total of 1070 published RCTs (median publication year: 2018; interquartile range: 2013-2020) were identified and tested. We found that 7.61% of all “low bias” (RoB 2)-rated RCTs were of high selection bias risk (FOR 7.61%; 95% CI: 6.31%-9.14%) and that the likelihood for high selection bias risk in “low bias” (RoB 2 domain 1)-rated RCTs was 6% higher than that for low selection bias risk (-LR: 1.06; 95% CI: 0.98-1.15). These findings raise issues about the validity of “low bias” risk ratings using Cochrane’s RoB 2 tool as well as about the validity of some of the results from recently published RCTs. Our results also suggest that the likelihood of a ”low bias” risk-rated body of clinical evidence being actually bias-free is low, and that generalization based on a limited, pre-specified set of appraisal criteria may not justify a high level of confidence that such evidence reflects the true treatment effect.

## Introduction

Randomized controlled trials (RCT) provide the most reliable information about the effectiveness of healthcare interventions [1], as long as there are no methodological flaws that would lead to the underestimation or overestimation of the true therapeutic effect (systematic error/bias) [2]. As a result, systematic reviews need to include bias risk assessment during trial appraisal. The Cochrane’s Risk of Bias (RoB) tool was developed for this purpose [3].

The first version of the tool was developed in 2005 based on an extensive list of potential sources of bias, extensive discussions, consultations, and testing, followed by agreement among statisticians, epidemiologists, and review authors [3].

The tool became widely used, and a second, updated version (RoB 2) was presented in 2019 [4]. The RoB 2 consists of 22 "signaling" questions concerning five bias domains about (i) randomization process, (ii) deviation from intended interventions, (iii) missing outcome data, (iv) measurement of outcome, and (v) selection of the reported result. During the trial appraisal, the tool offers the following response options to these questions: "low risk of bias," "high risk of bias," and "other" to be rated as "yes," "probably yes," "probably no," "no," "not applicable," or "no information." Following such assessment for each signaling question, the overall bias risk of an RCT is labeled either as “low,” “some concern,” or “high.” If all five bias domains of the RoB 2 tool have been judged as of “low bias” risk, then the entire RCT is overall rated as of “low bias” risk. If one domain is judged as of “high bias” risk or if “some concerns” are raised for a multiple number of domains, then the RCT is overall rated as of “high bias” risk [4].

To date, only poor inter-rater reliability has been established for the RoB 2 tool (inter-rater reliability (IRR): Fleiss’ Kappa 0.16; 95% CI: 0.08-0.24) [5]. The tool has also been described as complex and demanding, requiring intensive training and pilot runs before it can be correctly applied in any systematic review [5]. Despite an intense 40-hour rater-calibration process over three months, the overall IRR remained moderate (IRR = 0.42) [6].

Furthermore, the RoB 2’s empirical evidence based on a systematic review of meta-epidemiological studies supports only five out of its 22 signaling questions, namely whether group allocation was random (7% overestimation of trials with inadequate or unclear randomization: ROR: 0.93, 95% CI: 0.86-0.99, I^2^ = 0%) and whether the allocation sequence was concealed (10% overestimation of trials with inadequate or unclear allocation concealment: ROR: 0.90, 95% CI: 0.84-0.97, I^2^ = 28%). The evidence also supports questions in the domain "measurement of the outcome" in support of the blinded assessment of susceptive outcomes and double-blinding (23% overestimation of trials with inadequate or unclear double-blinding: ROR: 0.77, 95% CI: 0.61-0.93). However, no empirical evidence was established supporting the questions for the bias domains "deviation from intended intervention," "missing outcome data," and "selective reporting" [7].

Despite these shortcomings, the RoB 2 tool assigns an overall “low bias” risk rating to trials that have been judged to be of “low bias” risk in all of its five single bias domains, even if any methodological trial error inside or outside of these domains may have biased RCT results. One such error, related to selection bias risk, may be determined for an RCT using a test that relies on the I^2^ point estimate in meta-analyses. Hicks et al. suggested that heterogeneity in baseline variables, common to all trials in a meta-analysis between RCTs, should always be zero, and any measured differences in baseline values between the groups should occur only by choice. This was explained on the basis that true random allocation of patients in RCTs ensures a balanced distribution of baseline characteristics in intervention groups [8]. Clark et al. [9] further stated that baseline variables do not share the causes of heterogeneity in outcome variables (for example, differences in populations or intervention characteristics), and thus the only plausible explanation for heterogeneity in baseline variables is randomization errors [9].

The absence of heterogeneity beyond the play of chance corresponds to a zero I^2^ point estimate (%) in a baseline data meta-analysis. Any baseline imbalances in one or more trials caused by non-random allocation of patients to intervention groups would deviate from such zero value, indicating that the meta-analysis result has been affected by selection bias [9]. It has further been established that the test accuracy of the I^2^-point estimate in baseline variable meta-analyses is not affected by trial number and sample size [10]. In line with these principles, Mickenautsch and Yengopal adopted this test method to identify potential selection bias risk in single RCTs [11].

The aim of our study, using the adopted I^2^ test version for single trials [11], was to establish the risk of selection bias in RCTs that were overall rated as being of “low bias” risk by the RoB 2 tool. This article was previously posted to the Research Square preprint server on June 13, 2024.

## Materials and methods

The methodology of this study was adapted from an earlier protocol version published online [12]. The applied changes from the original protocol are listed in Section 1 in the Appendix. The main changes are added criteria details for systematic review exclusion, the inclusion of a sensitivity and subgroup analysis, and the addition of the false omission rate (FOR) as an outcome measure. As much as applicable, this study is reported according to the PRISMA statement [13] (see Section 2 in the Appendix for the complete PRISMA checklist and flowchart).

A systematic literature search of systematic review reports

Systematic review reports that met all of the following criteria were included: systematic review of RCTs; RCT quality rated using the second version of Cochrane’s RoB tool (as recommended by Sterne et al., 2019 [4]); application of the RoB 2 tool indicated in report abstract; and includes at least one RCT rated as of overall “high bias” and one RCT rated as of “low bias” risk.

PubMed was searched until January 24, 2024, using the following string of search terms: (systematic review RoB 2 OR Cochrane RoB 2.0 OR Cochrane RoB 2) AND systematic review with the following set limits: article type = systematic review; publication date = 1 year.

One reviewer (SM) searched by screening citation titles and abstracts. Systematic review reports in line with the selection criteria were retrieved in full copy. A second reviewer (VY) independently verified the retrieved articles for eligibility. Disagreements were resolved via discussion and consensus.

Data extraction and management

The full references of each selected systematic review report were recorded, and an ID number was assigned to each. The following data was extracted from each report and recorded in an MS Excel file (Microsoft Corporation, Redmond, Washington): systematic review ID number, number of RCTs with overall “low bias” rating, number of RCTs with overall “high bias” rating, and full reference of all RCTs.

Systematic reviews found during this process were excluded if they did not meet the following criteria: include at least one high and one low bias risk rating for each RCT, report an overall bias risk rating for trials, report the appraisal results of the first RoB tool version instead of the RoB 2, clearly apply or report the appraisal result in line with the RoB 2 tool, make supplementary material with details of bias risk appraisal accessible online, appraise RCTs but not other types of studies, provide information about trial appraisal, publish the RoB 2 graph reporting the appraisal results for each RCT in a readable manner, or report the full references of the appraised trials.

One reviewer (SM) extracted and entered all data into an MS Excel sheet. A second reviewer (VY) verified all data entry for accuracy. Disagreements were resolved via discussion and consensus.

RCT extraction and test for selection bias risk

All citations of the reviewed RCTs were extracted from the selected systematic reviews. With the help of an experienced librarian, an attempt was made to retrieve all of the identified RCTs in full copy. One reviewer (SM) reviewed the full RCT reports for eligibility, in line with the following selection criteria: trial reference reported by systematic review, full traceable clinical trial report, two separate treatment groups included, treatment groups were randomized and not matched by reported baseline variables, baseline variables reported per treatment group, mean (SD) values and precise sample size (N) reported per group, and no duplicates/different report of the same trial.

A second reviewer (VY) independently verified the reviewed reports for eligibility. Disagreements were resolved by discussion and consensus.

All selected RCTs were tested for selection bias risk following the test method suggested by Mickenautsch and Yengopal [11]. From each RCT, details of the baseline variable "age" were extracted for one test group and one control group, including mean value, standard deviation (SD), and number of subjects (N). If "age" was not reported, another reported baseline variable was chosen. Where more than one test and/or control group was reported, data extraction was limited to the test and control groups with the most significant baseline variable differences. Where standard error (SE) has been reported instead of SD, the SE was converted to SD using the formula: $SD = SE \times \sqrt{N}$.

All trials that did not report mean (SD or SE) values were excluded. Trials that reported median values with either minimum-maximum or interquartile range (IQR) were later included in the sensitivity analysis.

For each trial, two “simulated comparator trials” (SCT) were generated (Appendix Section 3). Each SCT consisted of two parallel data columns entered into an MS Excel sheet - column 1: random allocation sequence for two groups, A and B; column 2: list of randomly selected values within the trial-specific age range, sorted in ascending order.

The total number of subjects combined for the test and control groups was set at N = 100 per group. The random allocation sequence in column 1 was generated by block-randomization (block size = 4 for two groups: A, B) using the “Sealed Envelope” online tool [14]. The ascending list of randomly selected values in column 2 was generated using an online random number generator [15]. The comprehensive version of the online generator was used to randomly select the values of the baseline variable for each subject with the following settings: lower limit = 8/upper limit = 80; numbers to be generated = 200; allow duplication of results = yes; sort the results = yes/ascend; and type of result to generate = integer. Data column 2 was sorted according to allocation to groups A and B in column 1 using the sorting function in MS Excel. This process was repeated separately for the two SCTs, with separate sequences generated for columns 1 and 2, respectively.

After sorting, the mean (SD) value for each of the two SCTs was calculated and entered, along with the sample size per group A and B, into a fixed effect meta-analysis using Review Manager (RevMan 5.0.24 software). The two SCTs were pooled using the inverse variance method, and the resulting zero I^2^ point estimate was confirmed (Appendix Section 3).

To test an RCT for selection bias risk, the mean (SD) value of the baseline variable and the sample size (N) per group were entered into the generated SCT meta-analysis, and the analysis was repeated. The resulting new I^2^ point estimate was recorded. This procedure was repeated for each RCT, separately throughout the study (Appendix Section 4).

If the I^2^ point estimate of the repeated meta-analysis was also 0%, the test result was considered negative, and no selection bias risk for the tested RCT was assumed. If the point estimate showed an I^2^ > 0%, the test result was considered positive, and the tested RCT was assumed to be at high risk of selection bias.

Main statistical analysis

Throughout testing, either true negative (TN) or false negative (FN) and true positive (TP) or false positive (FP) values for overall “low bias” and overall “high bias” risk (RoB 2) ratings, respectively, were established: TP = “high bias”-rated trials with positive test result (I^2^ > 0%); TN = “low bias”-rated trials with negative test result (I^2^ = 0%); FN = “low bias”-rated trials with positive test result (I^2^ > 0%); and FP = “high bias”-rated trials with negative test result (I^2^ = 0%).

From these, the false omission rate (FOR), defined as the ratio between FN and the sum of FN + TN results, reported in %, with a 95% CI was computed. Within the context of this study, the FOR (95% CI) was considered the probability for an RCT to have a high selection bias risk, given an overall “low bias” risk rating using the second version of Cochrane’s RoB tool. The FOR value of 0% indicates zero probability for a “low bias” risk (RoB 2) rated RCT to have a high selection bias risk. It also indicates that no FN ratings were established.

Sensitivity analysis

Sensitivity analysis was conducted by adding data from RCTs that reported median values with either a minimum-maximum (min/max) range or IQR. This was followed by the exclusion of data from RCTs with any baseline variable other than "age" from the main analysis.

Median values with a min/max range or IQR were converted into mean (SD) estimates following the methods by Hozo et al. [16] and Wan et al. [17], respectively.

Subgroup analysis

Subgroup analysis was conducted for RCTs that were included in the main analysis and reported either a “low bias” or “high bias” risk rating for RoB 2 domain 1 concerning “bias arising from the randomization process”. Similar to the main analysis, either TN or FN and TP or FP values for “low bias” and “high bias” risk (RoB 2) ratings, respectively, were established using the same definitions as applied in the main analysis related to the established I^2^ values.

From the data, the -LR with 95% CI was computed. In line with convention [18], the -LR in this study was adopted as the ratio of the likelihood of an RCT rated as “low bias” risk for RoB 2 domain 1 to have high selection bias risk (I^2^ > 0%) divided by the likelihood of an RCT rated as “low bias” risk for RoB 2 domain 1 to have low selection bias risk (I^2^ = 0%).

The computed -LR (95% CI) was interpreted as the likelihood of an RCT rated as “low bias” risk for RoB 2 domain 1 to actually have low selection bias risk: -LR < 0.1 = highly likely, 0.1-0.2 = moderately likely, 0.2-0.5 = somewhat likely, and 0.5-1.0 = rarely likely [19].

A negative likelihood ratio (-LR) close to 1.0 indicated that an RCT rated as a “low bias” risk for RoB 2 domain 1 was almost more likely to have a low selection bias risk than a high selection bias risk, and -LR larger than 1.0 indicated that the risk of selection bias for such RCT was more likely to be high than low.

## Results

A systematic literature search of systematic review reports and RCT extraction

**Figure 1:**
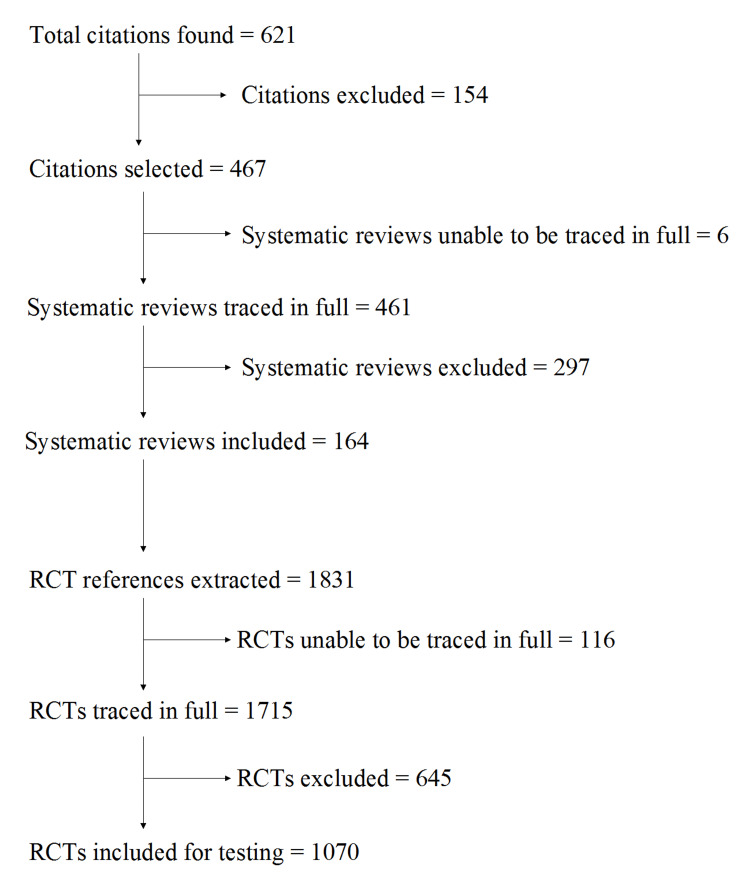
Flow diagram of the trial selection process RCT: Randomized controlled trials.

The systematic literature search identified 621 citations. Of these, 154 citations were excluded as not relevant, and 467 systematic review citations were included. Six systematic reviews could not be retrieved in full copy, and 461 systematic reviews were traced in full. Of these, 297 systematic reviews were excluded (Appendix Section 5). Reasons for exclusion were: high- and low bias risk ratings for at least one RCT, each; not included = 221; no overall bias risk rating reported for trials = 39; the appraisal results of the first RoB tool version were reported = 20, although the application of RoB 2 was reported in the abstract and/or methodology of the systematic review; the second version of the RoB tool was not applied or the appraisal result reported = 5; supplementary material with details of bias risk appraisal was not accessible online = 4; the systematic review did not appraise RCTs but other types of studies = 3; no information about trial appraisal was made available online in the report = 2; the published RoB 2 graph that reported the appraisal results for each RCT was unreadable = 2; and the appraised trials were not referenced in full = 1 (Figure 1).

A total of 164 systematic reviews were included for trial extraction. From these, 1831 RCT citations could be identified, and 1715 appeared to be retrievable in full copy. Of these, 645 were excluded. Reasons for exclusion were: no baseline variables reported per treatment group = 296; no mean (SD) values reported per group = 231; no trial reference reported by systematic review = 57; duplicates/different reports of the same trial = 23; no full trial report = 14; unable to be traced = 8; not two separate treatment groups included (split-mouth trial) = 7; treatment groups were not randomized = 3; treatment groups matched by reported baseline variables = 2; not a clinical trial = 2; no precise sample size (N) reported per group = 1; and data cannot be retrieved from trial = 1.

**Figure 2:**
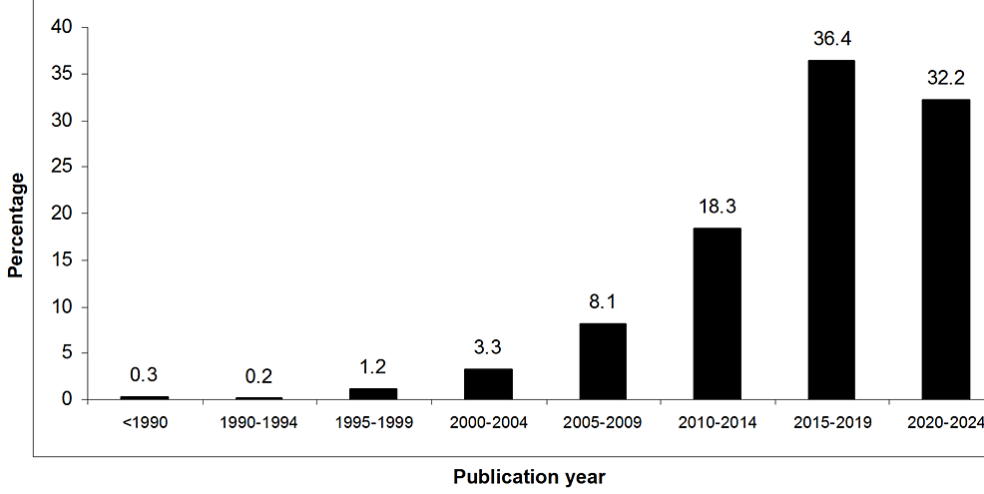
Percentage of published trials per publication year

**Figure 3:**
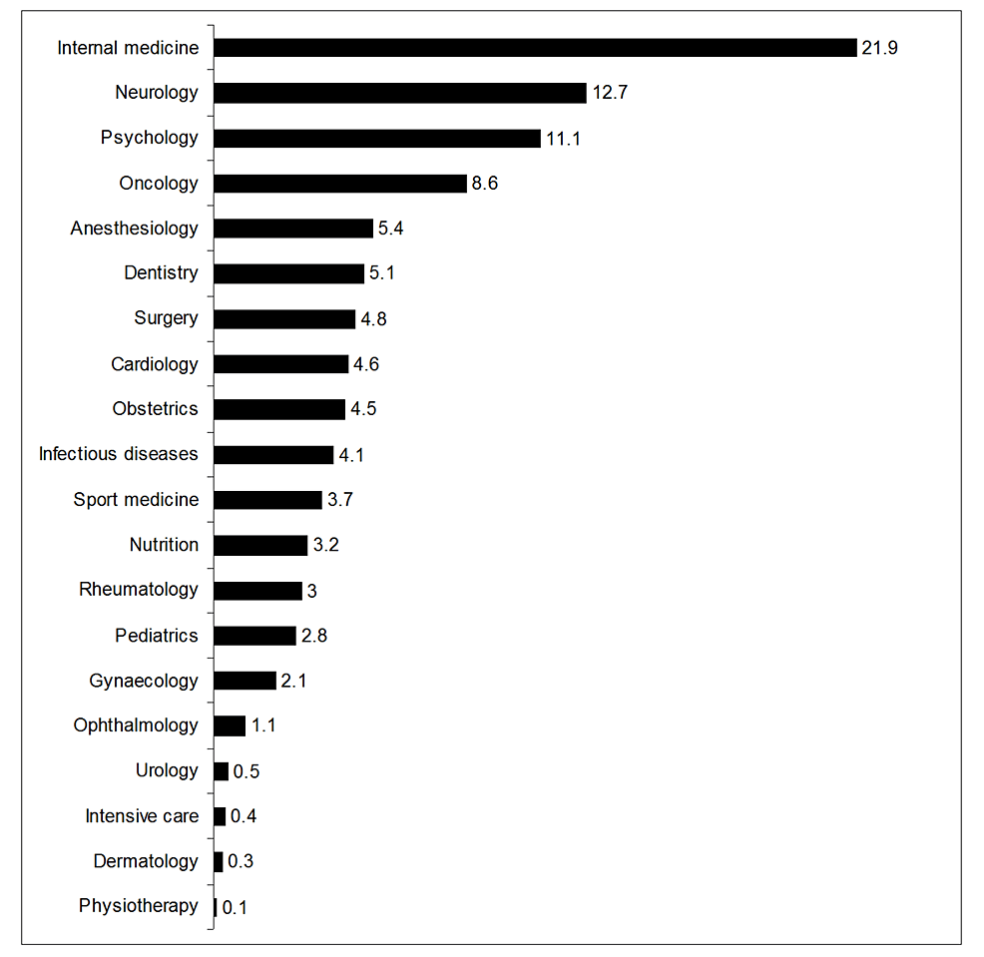
Percentage of trials per medical specialty

A total of 1070 RCTs were fully retrieved and tested for selection bias risk (Figure 1 and Appendix Section 6). These trials were published between 1977 and 2023, with 2018 (2013-2020) being the median (IQR) publication year (Figure 2). Most RCTs were published in the fields of internal medicine (21.59%), neurology (12.7%), and psychology (11.1%) (Figure 3).

Test for selection bias risk and main statistical analysis result

Testing of the 1070 trials yielded 562 TN, 46 FN, 37 TP, and 425 FP results. The total number of RCTs with high selection bias risk (I^2^ > 0%) was 83, resulting in a prevalence of high selection bias risk trials of 7.8% for this sample. The false omission rate was 7.61% (95% CI: 6.31%-9.14%) (Appendix Section 6).

Sensitivity analysis

When data from 140 RCTs reporting median values with either a minimum-maximum (min/max) range or IQR were added to the main analysis, the results changed to TN = 629, FN = 49, TP = 45, and FP = 487. The total number of RCTs with high bias risk (I^2^ > 0%) increased to 94, resulting in a prevalence of high bias risk trials of 7.8%. The false omission rate decreased to 7.26% (95% CI: 6.02%-8.73%).

When the data from 131 RCTs with any baseline variable other than "age" were excluded, the results changed to TN = 559, FN = 41, TP = 40, and FP = 438. The total number of RCTs with high bias risk (I^2^ > 0%) decreased to 81, resulting in a further decreased prevalence of high bias risk trials of 7.5%. The false omission rate decreased further to 6.82% (95% CI: 5.54%-8.37%) (Appendix Section 7).

Subgroup analysis

From the 1070 RCTs that were included in the main analysis, a “low bias” or “high bias” risk rating for RoB 2 domain 1 concerning “bias arising from the randomization process” could be ascertained for 768 trials (Appendix Section 8). These trials yielded 635 TN, 42 FN, 3 TP, and 88 FP results. The computed -LR was 1.06 (95% CI: 0.98-1.15).

## Discussion

Our study aimed to establish the risk of selection bias in RCTs that were rated as being of overall “low bias” risk using the RoB 2 tool, by applying an adopted I^2^ test version for single trials [11]. Accordingly, we established that 7.8% of RCTs in our sample of 1070 recent RCTs had high selection bias risk. A total of 7.61% of all RCTs rated overall as of “low bias” risk according to Cochrane’s RoB 2 tool were found to be of high selection bias risk (FOR: 7.61%; 95% CI: 6.31%-9.14%). Furthermore, the likelihood of an RCT, rated as of “low bias” risk for RoB 2 domain 1, having a high selection bias risk was 6% higher than having a low selection bias risk (-LR: 1.06; 95% CI: 0.98-1.15).

Main analysis results

Our study established a FOR of 7.61% (95% CI: 6.31%-9.14%), suggesting a 7.61% probability that an RCT with overall “low bias” risk rating using the RoB 2 tool is actually at high risk of having its trial results affected by selection bias. Such high risk is reflected by an established I^2^ point estimate above zero, indicating the existence of heterogeneity of a baseline variable ("age" in most of the tested trials) between the tested RCT and the two homogeneous SCTs that were included in the baseline variable meta-analysis. Such heterogeneity has been ascribed solely to problems with the randomization process [9].

The high selection bias risk was established based on data analysis and not by traditional trial appraisal methods for selection bias risk based on "text analysis," that is judging from the text of trial reports whether certain reported methodological trial characteristics (or their lack thereof) are in line with criteria that are conducive to low bias risk and pre-specified in trial appraisal tools, such as the RoB 2. Such judgment is empirically based on the results of meta-epidemiological studies that statistically compare the intervention effect estimates between trials with or without a certain trial characteristic (such as adequate randomization) [20]. A systematic review of meta-epidemiological studies established a statistically significant larger effect estimate for trials with "inadequate" or "unclear" allocation concealment (dSMD: 0.15; 95% CI: 0.03-0.28; I^2^ = 0%) as part of the randomization process compared to trials where it was judged to be "adequate" [21]. In this context, it is important to highlight that the established FOR of 7.61% (95% CI: 6.31%-9.14%) reflects that selection bias risk in RCTs is judged to have supposedly applied "adequate" randomization. This suggests that the actual effect estimate for trials with "inadequate" or "unclear" allocation concealment [21] may even be larger, thus indicating an even greater overestimation of the actual effect estimates, when RCTs that are incorrectly judged as "adequate" are considered as "inadequate."

While the probability of 7.61% for an RCT with an overall “low bias” risk rating to be of high selection bias risk may appear trivial within our sample of 1070 trials, it still would affect the proclaimed validity of a rather large number of recently published (i.e., “modern”) RCTs with lowest bias risk ratings, when considering the steadily increasing volume of clinical intervention trials worldwide [22]. Hence, future research is needed to confirm that the results of our sample are representative.

Our findings raise additional concerns about the validity of RoB 2 low bias risk ratings in particular as well as the limitations of "text analysis" (in contrast to "data analysis" based or statistical) methods for clinical trial appraisal in general.

Sensitivity analysis results

When data from 140 RCTs that reported median values with either a minimum-maximum (min/max) range or IQR were added to the main analysis, the FOR decreased from 7.61% (95% CI: 6.31%-9.14%) to 7.26% (95% CI: 6.02%-8.73%), and when the data from 131 RCTs with any baseline variable other than "age" were excluded afterward, the FOR decreased further to 6.82% (95% CI: 5.54%-8.37%).

These results may suggest that mean (SD) estimates, converted from median values, by not representing the directly measured mean (SD) values, may mask the presence of FN results in this study and thus may bias both the FOR point estimate and its CI toward a lower value. On the other hand, adding the data from the 140 additional trials reduced the width of the FOR CI while maintaining a significant overlap that included the value of the original FOR point estimate from the main analysis, contributing to an increase in the precision of the results.

Furthermore, the decrease in FN results caused by the exclusion of data from baseline variables other than "age" may indicate that the use of a single variable decreases the chance of identifying trials with high selection bias risk. Although "age" has been described as a good and easy baseline variable to reflect patient misallocation in RCTs that should always be included for bias testing [8,9], Hicks et al. also recommended the use of more than one baseline variable for testing [8]. A true absence of selection bias in an RCT will ensure that no heterogeneity for any baseline variable beyond the play of chance (reflected as I^2^ = 0%) will occur. As a result, the inclusion of more baseline variables in the bias test increases the chance of a valid positive test result for a trial affected by selection bias. Against this background, the FOR presented in our main analysis may be regarded as a rather conservative estimate of the actual FOR for our sample, which may, in reality, be much higher.

Subgroup analysis results

By investigating the RoB 2 domain 1 separately, we excluded all other types of bias, except selection bias, that may affect an RCT. Paradoxically, a low-risk rating for RoB 2 domain 1 did not decrease the likelihood of high selection bias risk. Rather, it increased it by 6% (-LR 1.06; 95% CI: 0.98-1.15). This suggests that in our sample of relatively recently published RCTs (median publication year: 2018; IQR: 2013-2020), trial appraisal for selection bias risk using the RoB 2 domain 1 generated unreliable results.

Furthermore, our observations provide empirical evidence to the simulation-generated hypothesis that the likelihood of a ”low bias” risk-rated body of evidence being actually error-free is small, and the generalization from any limited, pre-specified set of appraisal criteria rarely justifies a high level of confidence that such evidence reflects the true treatment effect [23]. This hypothesis was generated based on 45 simulation trials to which 0-5 possible errors out of a total of 65 error domains were randomly assigned. The results suggested that error-free evidence was only 1.2 times more likely (-LR 0.84; 95% CI: 0.80-0.88) to be rated as a "low bias" risk than evidence containing some form of error [23]. In comparison to these hypothetical simulation results, the results of our study (-LR 1.06; 95% CI: 0.98-1.15) are not only confirmatory but also suggest that such likelihood might even be less.

Limitations

The main limitation of our study is the uncertain external validity of its results. Our trial sample is not a random selection from all PubMed-listed RCTs. Instead, it is based on a systematic literature search of all systematic reviews of RCTs listed in PubMed within the previous 12 months. We chose the 12-month time limit to identify the most recent systematic reviews with similar exposure time to Cochrane’s RoB 2 tool. From the chosen trial selection process and subsequent trial exclusion, our study generated a sample of 1070 RCT reports published relatively recently with a main focus on medical topics related to internal medicine, neurology, and psychology. The level of rigor for selection bias control methods may be affected by a general process of the diffusion of these methods into the different fields of medicine [24,25]. Therefore, samples of RCTs with distributions in other medical fields and publication years that differ from those of our trial sample may produce different appraisal results of selection bias risk. In addition, the I^2^ point estimate was argued to artificially increase with the sample size of the included trials [26], and an increasing systematic error was established when the number of included trials was too few [27]. However, a simulation study of 558 meta-analyses showed no effect of sample size and trial number on the accuracy of the I^2^ point estimate when used in baseline variable (instead of outcome) meta-analyses and when the cut-off point for high selection bias risk was set at I^2^ = 0% [10].

## Conclusions

Our study established that overall “low bias” risk (RoB 2)-rated RCTs had high selection bias risk, with a statistically higher likelihood that such risk was higher than low. These findings raise concerns about the validity of RoB 2 “low bias” risk ratings (and subsequently trial result validity) for recently published RCTs, in particular, and show the limits of "text analysis" (in contrast to "data analysis") methods for bias risk appraisal of clinical trials, in general. Our results also suggest that the likelihood of a ”low bias” risk-rated body of clinical evidence being actually error-free is small and that the generalization from any limited, pre-specified set of appraisal criteria may rarely justify a high level of confidence that such evidence reflects the true treatment effect.

## Appendices

All data are made fully available without restriction in Appendices (Sections 1-8) and can be freely downloaded via the following link: https://data.mendeley.com/datasets/bt2j7rf4w5/1.

## References

[1] Kleijnen J, Gøtzsche P, Kunz RH (1997). So what’s so special about randomisation?. Non-random Reflections on Health Services Research: On the 25th Anniversary of Archie Cochrane’s Effectiveness and Efficiency.

[2] Wood L, Egger M, Gluud LL (2008). Empirical evidence of bias in treatment effect estimates in controlled trials with different interventions and outcomes: meta-epidemiological study. BMJ.

[3] Higgins JP, Altman DG, Gøtzsche PC (2011). The cochrane collaboration's tool for assessing risk of bias in randomised trials. BMJ.

[4] Sterne JA, Savović J, Page MJ (2019). RoB 2: a revised tool for assessing risk of bias in randomised trials. BMJ.

[5] Minozzi S, Cinquini M, Gianola S, Gonzalez-Lorenzo M, Banzi R (2020). The revised cochrane risk of bias tool for randomized trials (RoB 2) showed low interrater reliability and challenges in its application. J Clin Epidemiol.

[6] Minozzi S, Dwan K, Borrelli F, Filippini G (2022). Reliability of the revised cochrane risk-of-bias tool for randomised trials (RoB2) improved with the use of implementation instruction. J Clin Epidemiol.

[7] Page MJ, Higgins JP, Clayton G, Sterne JA, Hróbjartsson A, Savović J (2016). Empirical evidence of study design biases in randomized trials: systematic review of meta-epidemiological studies. PLoS One.

[8] Hicks A, Fairhurst C, Torgerson DJ (2018). A simple technique investigating baseline heterogeneity helped to eliminate potential bias in meta-analyses. J Clin Epidemiol.

[9] Clark L, Fairhurst C, Hewitt CE (2014). A methodological review of recent meta-analyses has found significant heterogeneity in age between randomized groups. J Clin Epidemiol.

[10] Mickenautsch S, Yengopal V (2024). Trial number and sample size do not affect the accuracy of the I2-point estimate for testing selection bias risk in meta-analyses. Cureus.

[11] Mickenautsch S, Yengopal V (2024). A test method for identifying selection bias risk in prospective controlled clinical therapy trials using the I2 point estimate. Cureus.

[12] Mickenautsch S, Yengopal V (2024). Selection bias risk in randomised control trials rated as of “low bias” risk according to Cochrane’s Risk of Bias 2 tool (protocol) [PREPRINT]. Res Sq.

[13] Moher D, Liberati A, Tetzlaff J, Altman DG (2009). Preferred reporting items for systematic reviews and meta-analyses: the PRISMA statement. PLoS Med.

[14] (2023). Sealed envelope. https://www.sealedenvelope.com/simple-randomiser/v1/lists.

[15] (2023). Random number generator. https://www.calculator.net/random-number-generator.html.

[16] Hozo SP, Djulbegovic B, Hozo I (2005). Estimating the mean and variance from the median, range, and the size of a sample. BMC Med Res Methodol.

[17] Wan X, Wang W, Liu J, Tong T (2014). Estimating the sample mean and standard deviation from the sample size, median, range and/or interquartile range. BMC Med Res Methodol.

[18] Deeks JJ (2001). Systematic reviews of evaluations of diagnostic and screening tests. Systematic Reviews in Health Care: Meta‐Analysis in Context.

[19] Jaeschke R, Guyatt GH, Sackett DL (1994). Users' guides to the medical literature. III. How to use an article about a diagnostic test. B. What are the results and will they help me in caring for my patients? The Evidence-Based Medicine Working Group. JAMA.

[20] Moustgaard H, Jones HE, Savović J, Clayton GL, Sterne JA, Higgins JP, Hróbjartsson A (2020). Ten questions to consider when interpreting results of a meta-epidemiological study-the MetaBLIND study as a case. Res Synth Methods.

[21] Mickenautsch S, Rupf S, Miletić I, Yengopal V (2022). Extension of the composite quality Score (CQS) as an appraisal tool for prospective, controlled clinical therapy trials-a systematic review of meta-epidemiological evidence. PLoS One.

[22] (2024). Number of clinical trial registrations by location, disease, phase of development, age and sex of trial participants (1999-2022). https://www.who.int/observatories/global-observatory-on-health-research-and-development/monitoring/number-of-trial-registrations-by-year-location-disease-and-phase-of-development.

[23] Mickenautsch S, Yengopal V (2023). The limits of inductive reasoning for clinical evidence appraisal - a simulation study [PREPRINT]. Res Sq.

[24] Carter K, Scheffold AL, Renteria J (2023). Regulatory guidance on randomization and the use of randomization tests in clinical trials: a systematic review [IN PRESS]. Stat Biopharma Res.

[25] Grayling MJ, Dimairo M, Mander AP, Jaki TF (2019). A review of perspectives on the use of randomization in phase II oncology trials. J Natl Cancer Inst.

[26] Rücker G, Schwarzer G, Carpenter JR, Schumacher M (2008). Undue reliance on I(2) in assessing heterogeneity may mislead. BMC Med Res Methodol.

[27] von Hippel PT (2015). The heterogeneity statistic I(2) can be biased in small meta-analyses. BMC Med Res Methodol.

